# The Effect of Vagus Nerve Stimulation on Pain Severity Scores in Patients With Chronic Musculoskeletal Pain Syndrome: A Meta-Analysis

**DOI:** 10.7759/cureus.103313

**Published:** 2026-02-09

**Authors:** Zachary J Buchman, Josie C Lorea, Daniel P Oar, Dante DiSilvestro, Logan F Jay, Christopher R Lazo, Megan Clark

**Affiliations:** 1 Medicine, Lake Erie College of Osteopathic Medicine, Bradenton, USA; 2 Medicine, University at Buffalo Jacobs School of Medicine and Biomedical Sciences, Buffalo, USA; 3 Physical Medicine and Rehabilitation, West Virginia University School of Medicine, Morgantown, USA

**Keywords:** chronic pain, chronic pain management, musculoskeletal pain, pain on vas, systematic review and meta analysis, tavns, tcvns, vagus nerve stimulation, visual analog scale, vns

## Abstract

This meta-analysis aimed to systematically evaluate and synthesize existing evidence on the efficacy of transcutaneous vagus nerve stimulation (tVNS) in reducing pain severity, as measured by numerical pain scales, among adults with chronic musculoskeletal (MSK) pain. A comprehensive PubMed literature search was conducted through March 2025. Inclusion criteria comprised randomized controlled trials or clinical trials involving adults with chronic MSK pain (e.g., fibromyalgia, lupus, and osteoarthritis) that evaluated tVNS using pre- and post-treatment Visual Analog Scale (VAS) or Numeric Rating Scale (NRS) pain scores. Six eligible studies were included. Data extraction encompassed study design, participant demographics, intervention parameters, and VAS/NRS outcomes. Statistical analyses were performed using both common-effects and random-effects models (REM), with heterogeneity assessed using I² and Tau² statistics. The findings support tVNS as a promising adjunctive treatment for individuals with chronic MSK pain. Across included studies, the pooled effect size demonstrated a significant mean improvement in pain severity from pre-treatment to post-treatment of 2.32 points (95% CI, 1.90-2.73) using the common-effects model and 2.23 points (95% CI, 0.31-4.15) using the REM. However, these findings are limited by the small number of included studies, small sample sizes, substantial heterogeneity related to variability in intervention parameters, and short follow-up durations. Accordingly, the results should be interpreted with caution. While tVNS appears to be a promising adjunctive therapy for chronic MSK pain, larger, well-controlled randomized trials are needed to establish its independent efficacy, optimal stimulation parameters, and safety.

## Introduction and background

Chronic musculoskeletal (MSK) pain syndromes, defined as persistent or recurrent pain affecting the bones, muscles, ligaments, or tendons that persists beyond the normal healing time of tissues (typically over three months), pose a significant and growing burden on global healthcare systems [1-4]. Chronic MSK pain can arise from heterogeneous etiologies, including degenerative causes such as osteoarthritis, inflammatory causes such as rheumatoid arthritis and lupus, or centrally mediated processes such as fibromyalgia [5,6]. The epidemiological burden of chronic pain is immense, with an estimated 30% of people worldwide experiencing chronic pain at some point in their lives [7]. However, not all demographics are affected by chronic pain equally, with a higher prevalence being reported in women, military veterans, people residing in rural areas, and individuals of lower socioeconomic status [7]. When measured by years lived with disability, chronic low back pain and neck pain consistently cause the greatest amount of disability globally, with other forms of chronic pain ranking closely behind [2]. In the United States alone, 50.2 million adults (20.5% of the US population) reported experiencing pain on most days or every day [8]. The socioeconomic costs are equally staggering, with an annual healthcare expenditure exceeding $150 billion in the US and €200 billion in Europe [3]. Even with this great burden, fewer than 2% of chronic pain sufferers ever attend a pain clinic for specialized treatment [3].

Despite advances in understanding chronic MSK pain syndromes, their management remains largely unsatisfactory [3]. In fact, two-thirds of all chronic pain sufferers report being dissatisfied with their current treatment [3]. The current standard of care includes pharmacological treatments such as nonsteroidal anti-inflammatory drugs (NSAIDs), anticonvulsants, skeletal muscle relaxants, antidepressants, and opioids [5]. Unfortunately, these treatments often provide only minimal to partial relief and are associated with significant adverse effects [5]. Non-pharmacological interventions, such as physical therapy, exercise, and psychological therapies, are also commonly used with some benefit but often fail to relieve severe or refractory pain [5]. An increasing number of interventional pain procedures are also being used, such as denervation surgery, nerve block injections, implantable drug delivery system implantation, and spinal cord stimulation device implantation [5]. However, nerve block injections can only be used with limited frequency, and other surgical treatments can often be too risky, invasive, or expensive for many patients [5]. The many limitations to the current management of chronic pain conditions necessitate clinicians to explore more effective and generalizable treatments with fewer adverse effects to adequately treat this large and unsatisfied patient population [5].

Vagal nerve stimulation (VNS) has emerged as a promising neuromodulatory intervention for various conditions, including epilepsy, depression, and stroke rehabilitation, in addition to recent exploration of its analgesic potential in chronic pain syndromes [5,9]. VNS involves the application of electrical impulses to the vagus nerve, either invasively via implanted devices or noninvasively through transcutaneous stimulation of the auricular or cervical branches [5,9,10]. The vagus nerve plays a central role in autonomic regulation and projects to multiple brain regions implicated in pain processing and modulation [5]. VNS has gained attention as a clinical modality aimed to reduce symptom severity in chronic pain syndromes [5]. Several different mechanisms have been hypothesized to be responsible for the analgesic effects of VNS [5,9]. First, VNS activates vagal afferents that inhibit spinal nociceptive reflexes, thus modulating ascending pain pathways and reducing pain perception [5,9]. Additionally, VNS exerts significant anti-inflammatory effects by modulating the connection between the nervous and immune systems to dampen the release of pro-inflammatory cytokines and promote the resolution of the inflammatory response [5,9,11,12]. VNS also influences the release of neurotransmitters such as serotonin and norepinephrine that are involved in descending pain inhibition, further contributing to its analgesic properties [5,13]. These mechanisms, in addition to others, are possible explanations for why a multitude of human and animal studies have demonstrated that VNS can increase pain thresholds, reduce somatic pain sensitivity, and improve subjective pain scores [5].

Despite the growing body of evidence supporting the analgesic efficacy of VNS in chronic MSK pain syndromes, the evidence remains fragmented and inconclusive. Studies vary widely in terms of patient populations, stimulation parameters, duration of treatment, and outcome measures, making it challenging to draw definitive conclusions about the effectiveness of VNS. While some trials report significant reductions in pain severity scores, others yield mixed or inconclusive results [14-19]. This emerging but inconsistent evidence base highlights the need for a rigorous systematic review and meta-analysis to synthesize the available data to inform clinical practice. A systematic review and meta-analysis will help clarify the therapeutic potential of VNS, guide future research directions, and ultimately improve pain management strategies for patients with chronic MSK pain syndromes.

## Review

Materials and methods

Research Question

Is there a significant difference in symptom severity scores before and after treatment with transcutaneous VNS in patients suffering from chronic MSK pain?

Inclusion Criteria

The study population included adult (18 years and older) males and females who suffer from chronic (more than three months) either generalized MSK or localized knee or low back pain. The studies utilized included both randomized controlled trials (RCTs) and clinical trials (CTs). The intervention being investigated was transcutaneous VNS, including both transauricular and transcervical modes of administration. The primary outcome was change in pain level before and after treatment utilizing the 10-cm Visual Analog Scale (VAS) (0-10) or an equivalent Numeric Rating Scale (NRS) (0-10). Only studies published in English were considered. As this systematic review and meta-analysis utilized previously published data to generate its conclusions, institutional review board approval was not required.

Exclusion Criteria

Studies unrelated to transcutaneous VNS for the treatment of chronic pain were excluded. Furthermore, studies that paired transcutaneous VNS treatment with other modalities were not included. Excluded study designs consisted of individual case reports, systematic reviews, and cross-sectional studies. To reflect contemporary clinical practice and current methodological standards, studies published before 2020 were not considered, and those that did not provide data regarding pre-treatment and post-treatment pain severity using VAS or an equivalent 0-10 scale were not included. Non-English studies were also excluded due to resource limitations preventing accurate translation and data extraction.

Search Strategy

**Figure 1:**
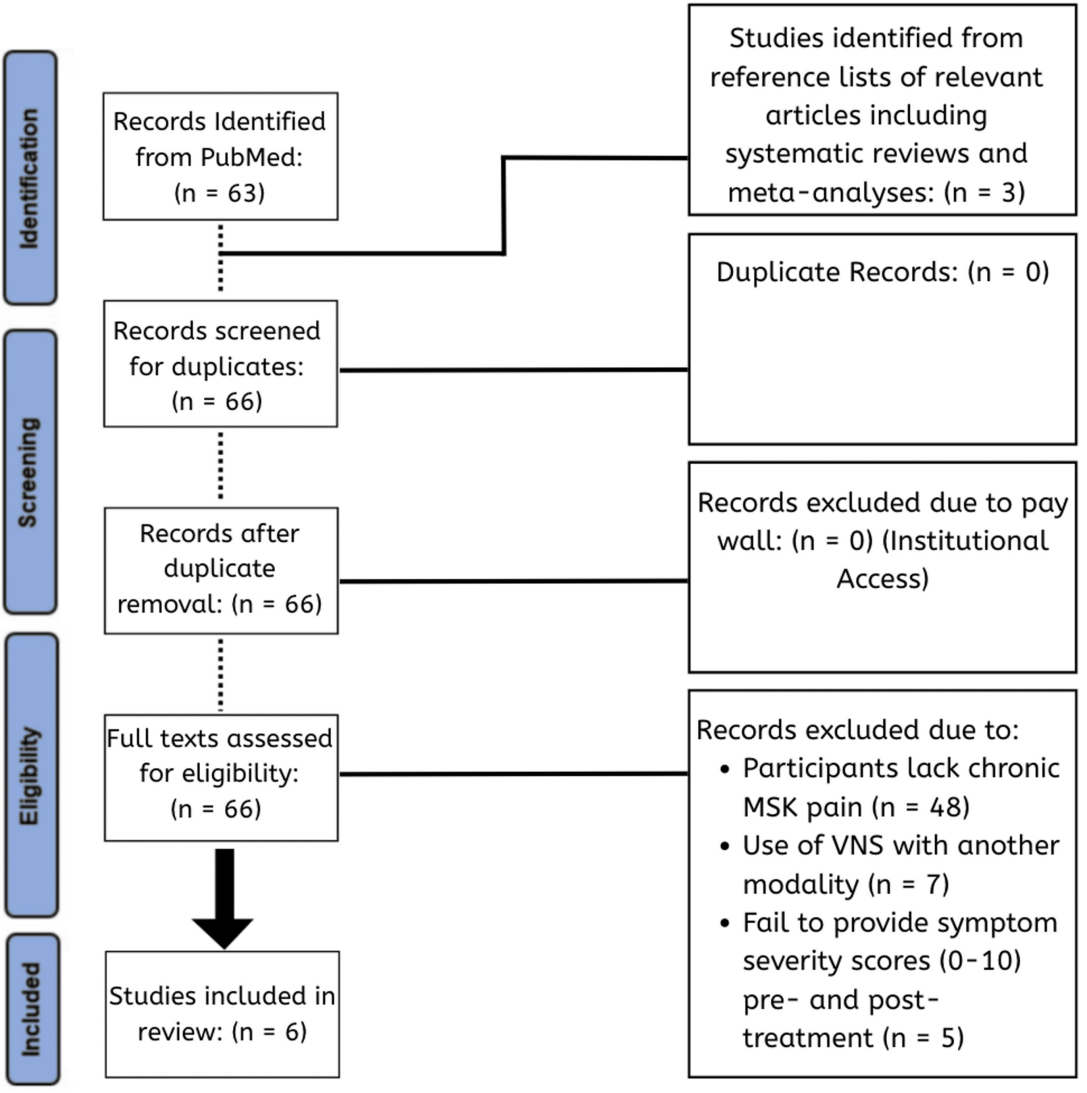
PRISMA flowchart demonstrating the literature search and study selection process PRISMA: Preferred Reporting Items for Systematic Reviews and Meta-Analyses, VNS: vagus nerve stimulation, MSK: musculoskeletal.

The Preferred Reporting Items for Systematic Reviews and Meta-Analyses (PRISMA) criteria framework was followed in the process of article selection [20]. A systematic search of the PubMed database was conducted through March 2025 using predefined Boolean search terms: (("Vagal Nerve Stimulation"[All Fields] OR "VNS"[All Fields] OR "Vagus Nerve Stimulation"[All Fields]) AND ("Chronic Pain"[All Fields] OR "Chronic Pain Syndromes"[All Fields] OR "Musculoskeletal Pain"[All Fields])) AND (2020:2026[pdat]). This search resulted in 66 results, which were subsequently screened in a sequential manner (Figure 1). No formal protocol was registered for this review.

Data Sources

A search for relevant systematic reviews and meta-analyses was conducted to identify and isolate other studies not listed under our original search in March 2025.

Study Selection

No duplicates were discovered during the literature search and screening. Studies that were neither RCTs nor CTs and studies unrelated to chronic musculoskeletal pain symptoms or transcutaneous VNS treatment were not included. There were no disagreements between reviewers (Z.B. and J.L.). No modifications to these methods were made after reviewing study results.

Data Extraction

Information regarding study design, sample demographics, sample size, quality and quantity of intervention utilized, pre- and post-treatment VAS/NRS scores, standard deviation, and confidence intervals were extracted from each study. For studies lacking reported standard deviations or confidence intervals (CIs), these values were calculated as necessary. Data reported on a 100-mm VAS were converted to a 10-cm scale for consistency. Data extraction was undertaken by authors Z.B., J.L., C.L., and L.J. Four researchers participated in this, and all work was double-checked. 

Quality Assessment

Bias was assessed using the NIH Study Quality Assessment Tool (NSQAT) and statistical analysis [21]. Only RCTs and CTs were used to minimize bias while maximizing study power. Further discussion of this process is described in the "Publication Bias" portion of this manuscript.

Statistical Analysis

**Figure 2:**
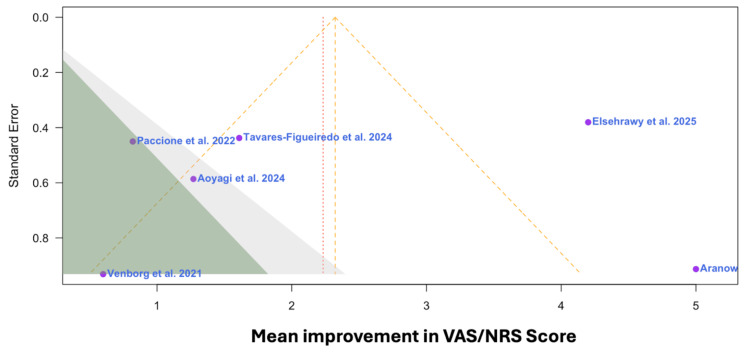
Funnel plot of the publication bias for all six studies included in this analysis Source: [14-19] VAS: Visual Analog Scale, NRS: Numeric Rating Scale.

**Figure 3:**
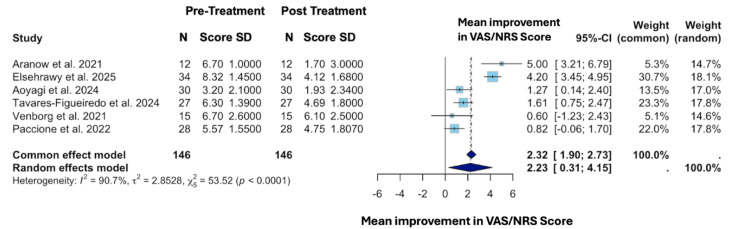
Forest plot demonstrating mean change in pain assessment using a Visual Analog Scale or Numeric Rating Scale for pre-treatment vs post-treatment with VNS Source: [14-19]. VAS: Visual Analog Scale, NRS: Numeric Rating Scale, VNS: vagus nerve stimulation, SD: standard deviation.

The funnel and forest plots in Figure 2 and Figure 3, respectively, were produced using RStudio version 4.4.1 (Posit, Boston, MA) and the meta package version 7.0-0. The funnel plot was generated to demonstrate each study's risk for publication bias, and the forest plot was generated to present each study’s mean change in symptom severity over the course of treatment and their associated confidence intervals. Although fewer than 10 studies were included, the funnel plot is presented as a descriptive tool to illustrate the distribution of effect sizes across studies. It is not intended for formal assessment of publication bias due to limited power. Through utilization of 95% CIs, the random-effects model (REM) investigated for possible heterogeneity between studies. Tests for heterogeneity were performed using the I^2^ and Tau^2 ^statistics to analyze variation among studies.

Assessment of Results

This meta-analysis includes calculations using both the common-effects model (CEM) and REM. When interpreting a meta-analysis with a large I^2 ^value indicating great heterogeneity, it is generally useful to analyze the REM [22]. Nonetheless, when limited by a relatively small to moderate number of studies incorporated into the meta-analysis, making it difficult to obtain an accurate estimate of between-studies variance, the CEM may be of benefit as well [23]. Due to the aforementioned reasons, a discussion of both models is included in the following sections.

Publication Bias

It is possible that bias played a role in limiting our search as the only manuscripts included focused on transcutaneous VNS related to the reduction of pain using a VAS/NRS. The NSQAT was implemented for each study to identify bias associated with the RCTs and CTs included in this study [21]. Two reviewers (L.J. and C.L.) conducted the quality assessment independently. For any responses to the provided questions resulting in disagreement, which occurred on greater than one instance, a third researcher (Z.B.) determined a tiebreaker judgement. The included studies were granted one point for each "yes" (Y) response and zero points for every "no" (N) response to the questions provided by the NSQAT, subsection “Guidance for Assessing the Quality of Before-After (Pre-Post) Studies with No Control Group” [21]. Final determination of risk of bias was made using the following scale: 0-3 points = “poor,” 4-8 points = “fair,” and 9-12 points = “good” [21]. Furthermore, the funnel plot displayed in Figure 2 demonstrates the individual studies' assessment for their respective risk of publication bias. Given the small number of studies, this funnel plot should be interpreted descriptively rather than as a formal test for publication bias.

Results

Literature Search

The initial literature search yielded 66 studies, of which 6 met the eligibility criteria for inclusion (Figure 1). These six studies (three RCTs [14,15,19] and three CTs [16-18]) investigated various forms of chronic MSK pain, including generalized pain symptoms [14,18,19] as well as localized pain symptoms [15-17]. All studies utilized comparable symptom severity scales (VAS/NRS) before and after treatment, solely with transcutaneous VNS.

Characteristics of the Included Studies

Six studies [14-19] were included in this meta-analysis. A summary of their individual characteristics is provided in Table 1.

**Table 1:** Summary of literature search results that were included in this statistical analysis RCT: randomized controlled trial, CT: clinical trial, MSK: musculoskeletal, SLE: systemic lupus erythematosus, VNS: vagus nerve stimulation, VAS: Visual Analog Scale, NRS: Numeric Rating Scale.

Study	Design	Treatment group size	Condition investigated	Intervention description	Pain scale measure
Aranow et al. (2021) [14]	RCT	12	MSK pain secondary to SLE	Five minutes of transcutaneous auricular VNS, once per day, for four consecutive days. 30 Hz frequency, 300 μs pulse width using Roscoe TENS 7000® device	VAS (0-10)
Elsehrawy et al. (2025) [15]	RCT	34	Knee osteoarthritis	Thirty minutes of transcutaneous auricular VNS, once per day, three days per week, for 12 consecutive weeks. 25 Hz frequency, 250 μs pulse width using Roscoe TENS 7000® device	VAS (0-10)
Aoyagi et al. (2025) [16]	CT	30	Chronic knee pain	One session of 60 minutes transcutaneous auricular VNS. 25 Hz frequency, 250 μs pulse width, 30 s on/off cycle using tVNS® device	NRS (0-10)
Tavares-Figueiredo et al. (2024) [17]	CT	27	Chronic low back pain	Thirty minutes of transcutaneous auricular VNS, once per day for three consecutive months. 25 Hz frequency, 50 μs pulse width using VAGUSTIM® device	VAS (0-100)
Venborg et al. (2021) [18]	CT	15	Polymyalgia rheumatica	Two minutes of transcutaneous VNS bilaterally on the neck three times daily for five consecutive days. 5000 Hz sine-wave pulses repeated at a rate of 25Hz using gammaCore® device	VAS (0-10)
Paccione et al. (2022) [19]	RCT	28	Fibromyalgia	Fifteen minutes of transcutaneous auricular VNS, twice daily for 14 consecutive days. 25 Hz frequency, 250 μs pulse width using Nemos® device.	NRS (0-10)

Risk-of-Bias Assessment

The risk of bias was assessed using the NSQAT, “Before-After (Pre-Post) Studies with No Control Group” [21]. The risk of biases in Aranow et al., Elsehrawy et al., Aoyagi et al., Tavares-Figueiredo et al., and Paccione et al. was determined to be “good,” and the risk of bias in Venborg et al. was determined to be “fair” [14-19] (Table 2). The greatest score (least risk for bias) was given to Paccione et al., which received a 10/12, and the lowest score (greatest risk for bias) was given to Venborg et al., which received an 8/12.

**Table 2:** Risk-of-bias quality assessment using the NSQAT “Before-After (Pre-Post) Studies With No Control Group” criteria questions Items 1-12 represent the 12 criteria questions used in the NSQAT “Before-After (Pre-Post) Studies With No Control Group” studies, which measure methodological quality of studies. Each question is answered with a "yes" or "no" [21]. G: good, F: fair, P: poor, Y: yes, N: no.

Study	Bias ruling	Score (out of 12)	#1 Was the study question or objective clearly stated?	#2 Were eligibility/selection criteria for the study population pre-specified and clearly described?	#3 Were the participants in the study representative of those who would be eligible for the test/service/intervention in the general or clinical population of interest?	#4 Were all eligible participants who met the pre-specified entry criteria enrolled?	#5 Was the sample size sufficiently large to provide confidence in the findings?	#6 Was the test/service/intervention clearly described and delivered consistently across the study population?	#7 Were the outcome measures pre-specified, clearly defined, valid, reliable, and assessed consistently across all study participants?	#8 Were the people assessing the outcomes blinded to the participants' exposures/interventions?	#9 Was the loss to follow-up after baseline 20% or less? Were those lost to follow-up accounted for in the analysis?	#10 Did the statistical methods examine changes in outcome measures from before to after the intervention? Were statistical tests done that provided p-values for the pre-to-post changes?	#11 Were outcome measures of interest taken multiple times before the intervention and multiple times after the intervention (i.e., did they use an interrupted time-series design)?	#12 If the intervention was conducted at a group level (e.g., a whole hospital, a community, etc.) did the statistical analysis take into account the use of individual-level data to determine effects at the group level?
Aranow et al. (2021) [14]	G	9	Y	Y	Y	Y	N	Y	Y	Y	Y	Y	N	N
Elsehrawy et al. (2025) [15]	G	9	Y	Y	Y	Y	Y	Y	Y	N	Y	Y	N	N
Aoyagi et al. (2025) [16]	G	9	Y	Y	Y	Y	Y	Y	Y	N	Y	Y	N	N
Tavares-Figueiredo et al. (2024) [17]	G	9	Y	Y	Y	Y	Y	Y	Y	N	Y	Y	N	N
Venborg et al. (2021) [18]	F	8	Y	Y	Y	Y	N	Y	Y	Y	Y	N	N	N
Paccione et al. (2022) [19]	G	10	Y	Y	Y	Y	Y	Y	Y	Y	Y	Y	N	N

The funnel plot displayed in Figure 2 demonstrates the heterogeneity of the studies used and the risk of publication bias across the individual selected studies [14-19]. The study closest to midline by Tavares-Figueiredo et al. [17] represents the study with the lowest risk of bias or variability; on the other hand, the study furthest away from the midline by Aranow et al. [14]** **represents the highest risk of bias or variability (Figure 2). The lowest data point on the plot by Venborg et al. represents the study with the smallest effect size (Figure 2) [18]. 

Findings

This meta-analysis included three RCTs and three CTs. Across these six trials, the total number of participants (N) was 146, all of whom received transcutaneous VNS and reported MSK pain severity on a scale of 0-10 before and after treatment. The overall effect size for all the studies using the CEM revealed a mean significant improvement in terms of symptom severity on VAS/NRS by 2.32 points (1.90; 2.73). Also, the overall effect size for all the studies using the REM revealed a mean significant improvement in terms of symptom severity on VAS/NRS by 2.23 (0.31; 4.15). Both the REM and CEM demonstrated a significant reduction in pain severity from pre- to post-treatment with transcutaneous VNS across all studies when all data were pooled (p < 0.0001). An I² value of 90.7% indicates substantial heterogeneity among the six included studies and rejects the hypothesis of no heterogeneity. The effect size of Aranow et al. was the greatest in terms of improvement in severity scores (average improvement of 5.00) but had a greatly reduced weight in comparison to the other included studies, with weights of 5.3% in CEM and 14.7% in REM [14]. The box sizes illustrated in Figure 3 are proportional to their weights, with Elsehrawy et al. having the highest weight in both the CEM (30.7%) and REM (18.1%) [15]. Furthermore, Elsehrawy et al. had the greatest precision with the smallest 95% CI range (3.45; 4.95) [15]. On the other hand, Venborg et al. had the lowest weight in both the CEM (5.1%) and REM (14.6%) [18]. Likewise, Venborg et al. had the least precision with the greatest 95% confidence interval range (-1.23; 2.43) [18].

Discussion

This meta-analysis investigated the efficacy of transcutaneous VNS in treating chronic MSK pain and found a statistically significant mean reduction of 2.32 points on VAS/NRS. This change exceeds the minimum clinically important difference (MCID) of 1.5-2.0 points for chronic pain conditions, indicating both statistical and clinical significance [24]. These findings suggest that transcutaneous VNS may offer substantial analgesic benefits for select patients.

The REM was employed to account for variability in study design, patient populations, and intervention protocols. This approach was particularly appropriate given the high degree of heterogeneity observed (I² = 90.7%), which indicates substantial inconsistency across studies. According to established thresholds, I² values above 75% suggest considerable heterogeneity, implying that the true effect size likely varies between trials [25]. This heterogeneity may stem from methodological differences, including variations in VNS modality (transcutaneous auricular VNS vs transcutaneous cervical VNS), stimulation parameters, treatment durations, and underlying patient diagnoses. As a result, while the pooled effect was statistically significant, interpretation must be approached with caution due to the high degree of variability between studies and the lack of a control group across the analyzed studies.

Substantial heterogeneity arose from differences in transcutaneous VNS modalities, stimulation protocols, and participant characteristics. For example, some trials used transcutaneous auricular VNS [14-17,19], while others employed transcutaneous cervical VNS [18]. These modalities vary in anatomical targeting, electrode placement, and potential engagement of vagal afferents, likely influencing clinical outcomes. There were also differences in stimulation frequency, intensity, and duration, ranging from a single 60-minute session to multi-week protocols [14-19]. Additionally, the underlying pain conditions varied widely, encompassing fibromyalgia [19], SLE-related pain [14], chronic low back pain [17], knee osteoarthritis [15], and polymyalgia rheumatica [18]. These conditions differ in their neuroinflammatory and central sensitization profiles, potentially moderating responsiveness to VNS [5].

Preliminary subgroup analysis suggests that patients with inflammatory pain, such as that from SLE or knee osteoarthritis, may respond more favorably than those with centrally mediated pain such as that due to fibromyalgia, consistent with the anti-inflammatory mechanism proposed for VNS [14,15,19,25]. However, due to the small number of studies per condition and limited statistical power, this observation should be considered hypothesis-generating rather than confirmatory.

Transcutaneous VNS is non-invasive, generally well-tolerated, and affordable relative to other neuromodulatory interventions [5]. Across included studies, adverse effects were rare and mild, typically limited to skin irritation or tingling at the stimulation site [14,19]. However, this treatment's non-pharmacologic nature can make it particularly more appealing for patients with contraindications to opioids or NSAIDs, or those suffering from the effects of polypharmacy. Moreover, transcutaneous VNS may positively influence social-emotional dimensions of chronic pain, which are often neglected in standard care [26]. Nevertheless, reporting of adverse events was inconsistent, and long-term tolerability remains understudied, raising concerns about underreporting bias and unknown delayed complications.

Risk-of-bias assessments using the NSQAT “Before-After Studies with No Control Group” tool revealed only slight variability in methodological rigor. Five of the six studies [14-17,19] were rated as “good,” while one [18] was rated as “fair,” primarily due to inadequate blinding, limited sample sizes, and short follow-up durations. Interestingly, studies with a higher risk of bias did not necessarily report a greater effect. For example, Venborg et al. [18], who had a “fair” rating, showed only modest improvement (VAS change of -0.6), while Paccione et al. [19], despite a “good” rating, also reported relatively small effects (VAS change of -0.8). This suggests that while bias may exist, it likely did not substantially skew the results toward overestimation.

The findings of this systematic review and meta-analysis are consistent with prior reviews on transcutaneous VNS for pain. A systematic review by Yap et al. on non-invasive VNS and its challenges for translation to clinical practice concluded that transcutaneous VNS can be an effective way to modulate the central nervous system in some cases [27]. In contrast, another systematic review by Eid et al. conducted on the effects of transcutaneous VNS on chronic low back pain found insufficient evidence to support the use of VNS alone as a viable therapeutic rehabilitation strategy [28]. Despite differing conclusions, both reviews highlight the therapeutic potential of transcutaneous VNS and emphasize the need for long-term controlled studies to determine true effectiveness, optimize treatment protocols, and better evaluate disability, quality of life, and pain outcomes [28].

This systematic review and meta-analysis reinforces a growing clinical interest in transcutaneous VNS as part of multimodal chronic pain management but does not yet warrant its incorporation into formal guidelines. The evidence base remains early-stage, with heterogeneity and methodological limitations preventing firm conclusions. Several key limitations must be noted. First, the number of included studies was small (six), and most had limited sample sizes, which reduces statistical power and increases the risk of type II error. Second, there was substantial heterogeneity in transcutaneous VNS delivery mode, dose, and treatment duration, hindering the identification of optimal stimulation protocols. Third, follow-up periods were generally short, making it difficult to assess the durability of treatment effects. Fourth, the risk of publication bias is significant. Fifth, the likelihood of underreporting of adverse effects across studies raises concerns about the completeness of safety data. Lastly, although the patient population studied suffers from chronic pain, the lack of control groups within all of the analyzed studies limits the amount of pain relief that can be attributed to transcutaneous VNS solely. In summary, this systematic review and meta-analysis found encouraging, but preliminary, evidence that non-invasive vagus nerve stimulation can be used to reduce chronic musculoskeletal pain with efficacy, which exceeds MCID. However, the high heterogeneity, small sample sizes, lack of a control group, variable study designs, and short follow-up durations prove the need for larger, well-controlled, and more standardized trials. Until such data is available, transcutaneous VNS should be viewed as a promising adjunctive therapy rather than a first-line treatment for chronic pain.

## Conclusions

This study systematically evaluated the effects of tVNS on pain severity among adults with chronic MSK pain syndromes. Pooled pre-post analyses demonstrated a statistically and clinically meaningful reduction in pain scores across included studies. However, due to substantial heterogeneity, limited sample sizes, short follow-up durations, and inconsistent use of control groups, these findings must be interpreted with caution. However, several caveats are to be considered when interpreting these. First, the small number of included studies, limited sample sizes, large heterogeneity due to variability in intervention parameters, and short follow-up durations limit the application and generalizability of this paper's conclusions. Further, possible underreporting of adverse effects and potential publication bias of the included studies highlight the importance of caution when applying these results to broader clinical practice. While tVNS appears to be a promising adjunctive, non-pharmacologic intervention within multimodal chronic pain management, current evidence is insufficient to establish its independent efficacy or support widespread clinical adoption. Future research should prioritize larger, well-controlled randomized trials with standardized stimulation protocols, extended follow-up periods, and comprehensive reporting of both efficacy and safety outcomes to better define its role in chronic pain treatment.
